# The prevalence, incidence, and impact of narcolepsy and idiopathic hypersomnia in Taiwan: comparison between the National Health Insurance Research Claims Database and a hospital cohort database

**DOI:** 10.1093/sleep/zsaf132

**Published:** 2025-05-20

**Authors:** Yu-Shu Huang, Wei-Chih Chin, I-Hang Chung, Tsun-Yi Roan, Chee-Jen Chang, Hsiao-Ting Juang, Shu-Chen Chang, Somraj Ghosh, Stephen Crawford, Huang-Li Lin

**Affiliations:** Division of Psychiatry and Sleep Center, Chang Gung Memorial Hospital, Taoyuan, Taiwan; College of Medicine, Chang Gung University, Taoyuan, Taiwan; Division of Psychiatry and Sleep Center, Chang Gung Memorial Hospital, Taoyuan, Taiwan; College of Medicine, Chang Gung University, Taoyuan, Taiwan; College of Life Sciences and Medicine, National Tsing Hua University, Hsinchu, Taiwan; Division of Psychiatry and Sleep Center, Chang Gung Memorial Hospital, Taoyuan, Taiwan; College of Medicine, Chang Gung University, Taoyuan, Taiwan; Division of Psychiatry and Sleep Center, Chang Gung Memorial Hospital, Taoyuan, Taiwan; College of Medicine, Chang Gung University, Taoyuan, Taiwan; Department of Artificial Intelligence, Chang Gung University, Taoyuan, Taiwan; Research Services Center for Health Information, Chang Gung University, Taoyuan, Taiwan; Department of Biomedical Sciences, Chang Gung University, Taoyuan, Taiwan; Department of Artificial Intelligence, Chang Gung University, Taoyuan, Taiwan; Research Services Center for Health Information, Chang Gung University, Taoyuan, Taiwan; Global Evidence and Outcomes – Neuroscience, Takeda Development Center Americas, Inc., Cambridge, MA, USA; Global Evidence and Outcomes – Neuroscience, Takeda Development Center Americas, Inc., Cambridge, MA, USA; Division of Psychiatry and Sleep Center, Chang Gung Memorial Hospital, Taoyuan, Taiwan; College of Medicine, Chang Gung University, Taoyuan, Taiwan

**Keywords:** narcolepsy type 1, narcolepsy type 2, idiopathic hypersomnia, National Health Insurance Research Database, epidemiology, disease impact

## Abstract

**Study Objectives:**

We investigated the prevalence and incidence of narcolepsy (types 1 and 2) and idiopathic hypersomnia (IH) in Taiwan using the National Health Insurance Research Database (NHIRD), and compared disease impact with patients identified from a “gold-standard” hospital database.

**Methods:**

This study employed retrospective cohort and cross-sectional designs to analyze data from the NHIRD and the hospital database cohort between 2009 and 2019. Analyses comprised prevalence and incidence of narcolepsy and IH, with diagnoses validated against the hospital database, and comparison of demographics, prescriptions, comorbidities, healthcare utilization, and costs with a control cohort. Categorical variables were analyzed using the chi-squared test; continuous variables were assessed via analysis of variance or Kruskal–Wallis tests.

**Results:**

From both databases, 24 317 patients were identified as having narcolepsy or IH. The diagnosed prevalence of narcolepsy was 9.98 per 100 000 individuals. Annual increases in prevalence and incidence were observed, particularly in young adults (aged 21–30 years). Patients with narcolepsy and IH exhibited higher rates of comorbidities and received more psychotropics compared with controls. However, fewer patients from the NHIRD received treatment for hypersomnolence than those from the hospital cohort. Healthcare utilization and costs were higher among patients with narcolepsy and IH compared with controls.

**Conclusions:**

Increased narcolepsy prevalence over time was observed, particularly among young adults, but the overall prevalence in Taiwan appears to be lower than in other countries, potentially indicating underdiagnosis or inadequate treatment. High comorbidity rates and healthcare utilization underscore the substantial disease impact in patients with central hypersomnia.

## Graphical Abstract

**Graphical Abstract:**
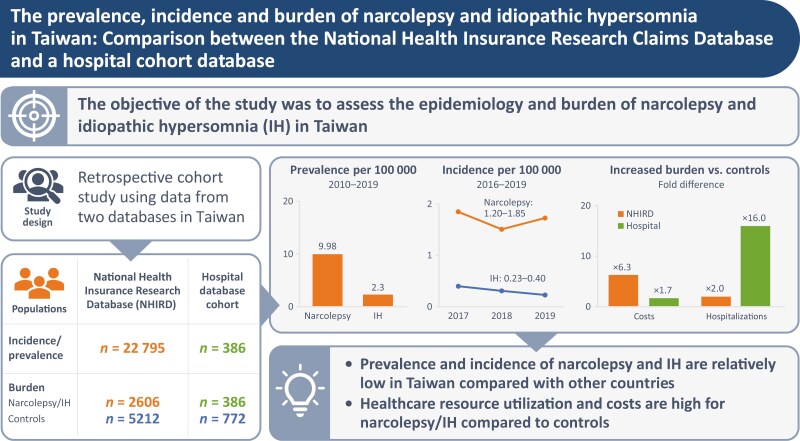

**Statement of Significance**

The prevalence of narcolepsy varies considerably. This study investigated the prevalence and incidence of narcolepsy and idiopathic hypersomnia (IH) in Taiwan using the National Health Insurance Research Database (NHIRD), comparing disease burdens with a hospital cohort database. Although annual prevalence and incidence suggested increasing trends over time, the diagnosed prevalence of narcolepsy was 9.98 per 100 000 individuals, lower than in other countries. Patients with narcolepsy and IH exhibited higher comorbidity rates and healthcare utilization and received more psychotropics than controls. However, fewer patients from the NHIRD received treatment for hypersomnolence than those from the hospital cohort. These findings indicate potential underdiagnosis of narcolepsy in Taiwan and highlight the need to improve treatment and management, considering the high disease burdens of central hypersomnia.

Narcolepsy is a rare, chronic central nervous system disorder of hypersomnolence characterized by excessive daytime sleepiness, which can be associated with cataplexy, hypnagogic or hypnopompic hallucinations, sleep paralysis, and disrupted nighttime sleep [1]. The International Classification of Sleep Disorders (ICSD), Third Edition, Text Revision (ICSD-3-TR) further classifies narcolepsy into type 1 (NT1) and type 2 (NT2) [2]. Patients with NT1 have cataplexy, a transient muscle tone loss often triggered by emotion, and/or orexin (hypocretin) deficiency (≤110 pg/mL, or less than one-third of mean values from individuals without narcolepsy), whereas patients with NT2 do not have cataplexy and have normal cerebrospinal fluid orexin levels [1]. The most widely accepted hypothesis for the pathophysiology of NT1 is an autoimmune mechanism causing the selective loss of orexin-releasing neurons within the lateral hypothalamus [3–5], with the resulting reduced orexin levels in the brain and cerebrospinal fluid causing the complex symptoms of narcolepsy [4–12]. The pathophysiology of NT2 remains elusive; however, it may be caused by a moderate loss of orexin neurons and/or other distinct factors [13, 14]. Idiopathic hypersomnia (IH) has a specific diagnostic definition in ICSD-3-TR, and is characterized by excessive daytime sleepiness, often with prolonged nighttime sleep, long unrefreshing naps, and difficulty waking up [2]; however, symptomatic overlap between IH and NT2 can make a differential diagnosis between the two disorders challenging [15].

The prevalence of narcolepsy or IH varies globally and can be influenced by different ethnicities. Reported prevalence estimates for narcolepsy span a wide spectrum, from the highest (590/100 000 people) in Japanese populations [16, 17] to the lowest (0.23/100 000) in Israeli Jews [18]. In the United States, Acquavella et al. (2020) reported prevalence estimates between 38.9 and 44.3 per 100 000 from 2013 to 2016 [19]. Meanwhile, in US claims database analyses, Abioye et al. (2022) reported a narcolepsy prevalence of 53.3 per 100 000 and Scheer et al. (2019) reported a prevalence of 79.4 per 100 000 [20, 21]. In a general population study in the United States, Ohayon et al. (2023) reported a narcolepsy prevalence of 37.7 per 100 000 people [22]. Asian studies have revealed prevalences of 0.015% (15/100 000) in Korea [23], 0.034% (34/100 000) in Hong Kong [24], and 0.0185% (18.5/100 000) to 0.0375% (37.5/100 000) in Japan [25, 26]. A systematic review of cohort or cross-sectional general population studies published up to 2021 reported prevalence estimates ranging from 1.05 to 79.4 per 100 000, underscoring the complexity and high heterogeneity of narcolepsy epidemiology [22, 27, 28]. Information on the prevalence of IH is less abundant; in the United States and Europe, estimates range from 7.8 to 14.6 per 100 000 people [19, 21], and a recent claims-based analysis reported a prevalence of 7.7 per 100 000 in Japan [26].

Even in the same geographic area, prevalence can vary across different studies. In Taiwan, two studies utilizing Taiwan’s National Health Insurance Research Database (NHIRD) have yielded inconsistent prevalence estimates, ranging from 0.0129% (12.9/100 000) [29] to 0.28% (280/100 000) [30]. The large variability between studies may be attributed to differences in study methodologies, necessitating further investigation of the validity of these estimates.

The disease impact of narcolepsy and IH extends beyond medical costs, encompassing comorbidities that include both physical and psychiatric disorders, which can significantly impact patients’ quality of life [15, 31, 32]. As both diseases generally start in the second decade of life, high disease impact can be found not only in adults but also in children and adolescents [15, 33–36]. The most common physical comorbidities of narcolepsy include dyslipidemia, endocrine disease, obesity, cardiovascular diseases, and hypertension, and the most common psychiatric comorbidities include major depressive disorder, dysthymia, bipolar disorder, panic disorder, agoraphobia, social anxiety, and attention-deficit/hyperactivity disorder (ADHD) [31, 32, 37, 38]. Moreover, co-occurring psychiatric disorders and psychological burdens, including depression, anxiety, suicidal thoughts and feelings of hopelessness, as well as sleep disorders such as obstructive sleep apnea (OSA) and insomnia, have also been reported [39, 40]. These impacts can further impair an individual’s ability to cope with stressful situations. Varallo et al. (2024) found that patients with NT1 developed poorer coping strategies, with evidence of less active coping and planning and more behavioral and mental disengagement than a control cohort [41]. Increased psychotropic use has also been reported in patients with narcolepsy experiencing psychiatric comorbidities, including selective serotonin reuptake inhibitors, anxiolytic benzodiazepines, hypnotics, serotonin-norepinephrine reuptake inhibitors, and tricyclic antidepressants [42]. Similarly, a higher prevalence of schizophrenia has been observed in patients with narcolepsy compared with controls [32, 43]. Frequently reported comorbidities of IH include autoimmune, autoinflammatory, and allergic disorders [44]; obesity [44, 45]; migraine [46]; ADHD [47, 48]; and depression and anxiety [49–51].

Due to the low prevalence of narcolepsy and IH, it can be difficult to recruit large numbers of patients in clinical settings, but the low positive predictive values of rare diseases necessitate huge sample sizes for epidemiological studies. Utilizing healthcare databases like the NHIRD offers valuable insights for clinical research and policy formulation. Their strength is their larger-scale and long-term documentation, but these databases can also have more errors, such as inaccuracy of diagnosis coding and missing data, which result in validity concerns [52]. Validation methodologies are imperative to mitigate potential data inaccuracies and to avoid misleading results. Taiwan’s NHIRD is derived from the National Health Insurance program. The program was implemented in March 1995 and provides mandatory universal health insurance for approximately 99% of more than 23 million people in Taiwan. In this study, we utilized a hospital cohort database from Chang Gung Medical Center [32, 53, 54] to corroborate findings of the NHIRD data using a sub-cohort of patients with narcolepsy in the NHIRD that also sought care at the Chang Gung Medical Center. This study aimed to elucidate the prevalence and incidence of narcolepsy and IH in Taiwan, as well as associated disease burden, including comorbidities, and psychotropic use, by comparing the two databases.

## Methods

### Study design

This retrospective cohort study used patient-level data from two different healthcare databases in Taiwan. The first database was the NHIRD of Taiwan, a comprehensive, population-based claims database, used for the evaluation of prevalence and incidence of narcolepsy and IH. The NHIRD encompasses personal characteristics alongside details of clinical information, including demographics, date, expenditures, inpatient and outpatient procedures, and prescriptions. Taiwan’s Ministry of Health and Welfare established the Health and Welfare Data Science Center (HWDC), a data repository site that centralizes the NHIRD and other health-related databases for data management and analyses. Individual observation periods commenced from the date of birth, date of first registration, or January 1, 2000, whichever was most recent, and ended on the date of death, date of first transfer out, or December 31, 2019, whichever occurred first. The study spanned from January 1, 2009, to December 31, 2019.

The second database, referred to as the hospital database cohort, originated at the Chang Gung Memorial Hospital in Taiwan and was used to validate diagnoses captured in the NHIRD. Data were collected from sleep clinics following participants’ provision of informed consent. Collected variables included demographic information, comorbidities, polysomnography (PSG), multiple sleep latency test (MSLT), and data of HLA typing and subjective questionnaires. Data collection spanned from January 1, 2009, to October 31, 2019 [32, 53, 54]. Both the NHIRD and hospital database cohorts were used for the evaluation of disease burden. This study was approved by the Institutional Review Board of Chang Gung Memorial Hospital (201902163A3, 202001975B0, 202202146A3).

### Patient identification

**Figure 1.**
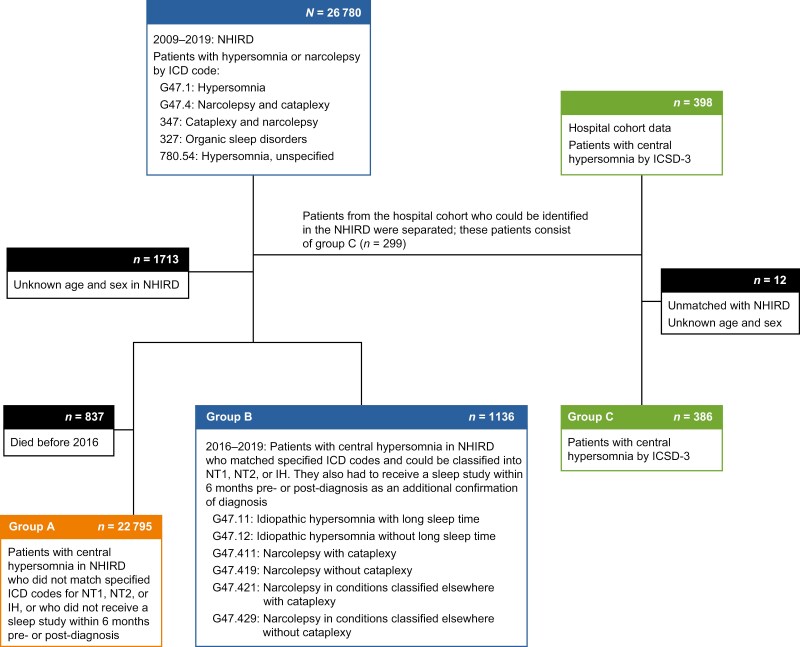
Screening flow chart for patients with central hypersomnia disorder identified in the NHIRD from 2009 to 2019, including those matched with patients from the hospital database cohort. Patients with ICD-9 codes (327, 347, or 780.54) and ICD-10 codes (G47.1 or G47.4) were included. Those with unknown sex or age were excluded. Group A (*n = *22 795) consisted of patients with central hypersomnia codes that could not be classified as narcolepsy type 1 (NT1), narcolepsy type 2 (NT2), or idiopathic hypersomnia (IH), or those who did not receive any sleep study 6 months before or after diagnosis. Group B (*n = *1136) consisted of those who could be classified as having NT1, NT2, or IH and received at least one sleep study (polysomnography or multiple sleep latency test) 6 months before and after the diagnosis. Once identified in the hospital cohort database, identification digits and ICD-9/ICD-10 codes were used to identify patients and their diagnoses in the NHIRD; patients for whom data did not match between the two databases or had unknown sex and/or age were excluded (*n = *12). Group C (*n = *386) consisted of patients with confirmed central hypersomnia diagnoses from the hospital cohort who were matched with patients from the NHIRD. ICD-9, International Classification of Diseases, Ninth Revision; ICD-10, International Classification of Diseases, 10th Revision; ICSD-3, International Classification of Sleep Disorders, Third Edition; NHIRD, National Health Insurance Research Database.

Patients were identified from the two databases (Figure 1). Inclusion criteria of the NHIRD comprised patients with (1) International Classification of Diseases, Ninth Revision, Clinical Modification (ICD-9-CM) codes, with 327, 347, or 780.54 assigned in one outpatient, emergency department, or inpatient event; or (2) International Classification of Diseases, 10th Revision, Clinical Modification (ICD-10-CM) codes, with G47.1 or G47.4 assigned in one outpatient, emergency department, or inpatient event (Supplementary Table S1). Those with unknown sex or age were excluded. Subgroups were delineated based on ICD codes. The presence or absence of sleep studies were used as an additional confirmation of diagnosis (PSG or MSLT; National Health Insurance code: 20044B). Group A consisted of those assigned ICD codes of central hypersomnia who could not be classified as having NT1, NT2, or IH, or those who did not receive any sleep study 6 months before and after the diagnosis (Figure 1). In order to precisely define patients, group B consisted of those who could be classified as having NT1, NT2, or IH and who received at least one sleep study 6 months before and after the diagnosis.

Inclusion criteria of the “gold-standard” hospital database cohort encompassed patients with a diagnosis of narcolepsy with cataplexy and narcolepsy without cataplexy per the ICSD, Second Edition (ICSD-2), diagnostic criteria (before 2016) [55]; NT1 (i.e. narcolepsy with cataplexy) and NT2 (i.e. narcolepsy without cataplexy) per the ICSD-3 diagnostic criteria (after 2016, when the ICSD-3 was adopted by the NHIRD of Taiwan) [1]; and IH per the ICSD-2 or ICSD-3 criteria (date dependent) [1, 56]. These patients received thorough clinical evaluations and examinations, including PSG, MSLT, actigraphy, HLA typing, or orexin (hypocretin) testing to confirm their diagnoses. Once identified in the hospital database cohort, identification digits and ICD-9/ICD-10 codes were used to identify patients and their diagnoses in the NHIRD. Those who could be identified in both databases were included in the hospital cohort (group C) and excluded from the NHIRD cohort (groups A and B); patients for whom data did not match between the two databases or had unknown sex and/or age were excluded from group C.

**Figure 2.**
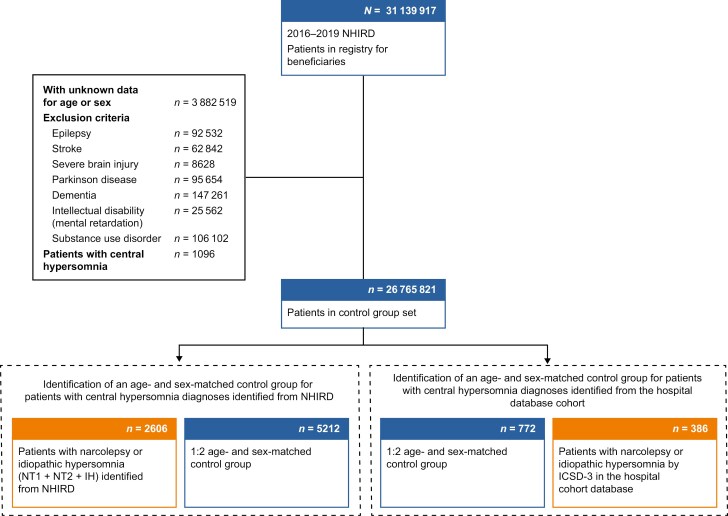
Identification of age- and sex-matched control groups for patients with central hypersomnia diagnoses identified from the NHIRD and the hospital database cohort. After excluding those with epilepsy, stroke, severe brain injury, Parkinson’s disease, dementia, intellectual disability, or substance use disorder, a total of 26 765 821 patients without central hypersomnia diagnoses were identified in the NHIRD. Of these, 5212 patients (2-fold the number of patients with central hypersomnia from the NHIRD [*n = *2606]) were age and sex matched to those identified with narcolepsy or IH from the NHIRD, and 772 patients (2-fold the number of patients with central hypersomnia from the hospital cohort [*n = *386]) were age and sex matched with the hospital database cohort, and were included as the control groups. ICSD-3, International Classification of Sleep Disorders, Third Edition; IH, idiopathic hypersomnia; NHIRD, National Health Insurance Research Database; NT1, type 1 narcolepsy; NT2, type 2 narcolepsy.

Age- and sex-matched control groups without a diagnosis of central hypersomnia were drawn from the NHIRD (Figure 2). The sample sizes of the control groups were twice the number of patients with central hypersomnia from the NHIRD and hospital database cohorts. Those with epilepsy, stroke, severe brain injury, Parkinson’s disease, dementia, intellectual disability, and substance use disorder were excluded.

### Data collection

In the NHIRD, data related to diagnoses were collected during 2009–2019, inclusive of a 1-year look-back period, to estimate prevalence and incidence during 2010–2019. Demographic data collected included age, sex, and birth history. Comorbidities were identified with at least three records during clinical visits and included diabetes mellitus, hypertension, hyperlipidemia, cardiovascular disease, obesity, autoimmune diseases, metabolic complications, epilepsy, anxiety disorders, major depression, dysthymia, bipolar disorder, ADHD, obsessive compulsive disorder, autism spectrum disorder (ASD), schizophrenia, and sleep disorders (OSA and other sleep disorders, such as insomnia). Prescription data collected included medications for hypersomnolence (only methylphenidate [MPH] and modafinil were available for treating hypersomnolence in Taiwan before 2020), medications for cataplexy (antidepressants), and comedication (antipsychotics, anxiolytics, hypnotics, and non-benzodiazepine hypnotics [Z-drugs]). Healthcare resource utilization data included hospitalizations and emergency department visits. All healthcare costs include inpatient and outpatient healthcare costs. Narcolepsy- and IH-related healthcare costs included costs of visits with narcolepsy/hypersomnia codes (including sleep studies, medication, and clinical evaluation). All costs were recorded in US dollars (USD) based on an exchange rate of 1 Taiwan dollar = 0.031 USD (January 21, 2025).

In the hospital database cohort, the index date was defined as the date of the first PSG and/or MSLT for narcolepsy diagnosis from outpatient records. A baseline period, extending 1 year prior to the index date, was utilized to assess the demographics of the study population (no index date was applied for the NHIRD analysis).

### Statistical analysis

In the NHIRD dataset, the prevalence and incidence rate of narcolepsy and IH each year were calculated as follows:


$$\begin{array}{l} Prevalence=\frac{Narcolepsy/IH\;diagnosed\;cases}{Population\;at\;31\;Dec\;of\;each\;year}\times \;100\;000 \end{array}$$

$$\begin{array}{l} Incidence\;rate=\frac{New\;narcolepsy/IH\;diagnosed\;cases}{Population\;\;at\;31\;Dec\;of\;each\;year}\;\times \;100\;000 \end{array}$$

All data management and statistical analyses were conducted using SAS version 9.4 at the HWDC. Quantitative variables were presented as descriptive statistics. Continuous variables were age and healthcare cost. Age was presented with mean and standard deviation and compared between groups using analysis of variance. Healthcare costs were presented as median and interquartile range. Positive skewness of healthcare utilization data was noted, and thus the data were compared between groups using Kruskal–Wallis tests. Categorical variables were presented as frequencies and percentages and were compared between groups using chi-squared tests. All tests were two-sided; a *p*-value <.05 was considered statistically significant.

## Results

### Distribution of patients in the NHIRD and the hospital cohort database

A total of 26 780 patient records with hypersomnia or narcolepsy ICD codes were retrieved from the NHIRD between 2009 and 2019. Of these, 23 931 were included in the study; 1713 were excluded owing to unknown age and sex, 837 were excluded owing to death before 2016, and 299 were excluded owing to identification in the hospital database (Figure 1). The NHIRD group A included 22 795 patients with unspecific central hypersomnia diagnoses or with no record of a sleep study conducted between 2009 and 2019, and the NHIRD group B included 1136 patients with an ICD-10 code for central hypersomnia (G47.411, G47.412, G47.411, G47.419, G47.421, or G47.429) and a sleep study 6 months before or after the diagnosis date (Figure 1). In the hospital database cohort, 398 patients with confirmed central hypersomnia diagnoses were identified and cross-referenced with NHIRD records by their identification digits. Twelve patients identified in the hospital cohort either did not match with NHIRD data or had unknown sex and/or age, leaving a total of 386 patients included in the hospital database cohort (group C).

There were significant differences in sex, age, and diagnosis distribution among the three groups (all *p* < .001; Table 1), suggesting high group heterogeneity. The mean (± standard deviation) age of group C was 23.7 ± 10.3 years, which was significantly younger than group B (29.7 ± 15.7 years) and group A (47.2 ± 19.2 years). There were significantly more males in group A than in groups B and C. In group C, there were more patients with NT1 than NT2 or IH; group B had more patients with NT2 than with NT1 or IH.

### The prevalence and incidence of NT1, NT2, and IH in Taiwan (NHIRD)

The prevalence of narcolepsy from both the NHIRD and the hospital databases was calculated for patients with unspecified central hypersomnia diagnoses excluding patients with IH, with or without a sleep study (22 529 from group A [22 795–266], 923 from group B [1136–213], and 386 from group C; *N = *23 771; Table 1), and was determined to be 100.82 per 100 000 people between 2009 and 2019 based on a yearly averaged Taiwan population of 23 575 774. In contrast, inclusion of only patients classified as having NT1 (*n = *939) and NT2 (*n = *1416; total *n = *2355; Table 1) resulted in a markedly lower narcolepsy prevalence of 9.98 per 100 000 people. The prevalence of IH between 2009 and 2019 was 2.3 per 100 000 people.

**Table 1.** Demographic Characteristics for Prevalent Cases of Central Hypersomnolence in the NHIRD and Hospital Database Cohorts Between 2009 and 2019

Characteristics	NHIRD		Hospital cohort	Total, *n* (*N* = 24 317)	*P*-value	Post hoc (groups)
	With 3 or 5-digit ICD codes or without a sleep study^*^ (group A, *n *= 22 795)	With identified ICD-10 codes and a sleep study^*^ (group B, *n* = 1136)	With confirmed diagnosis (group C, *n* = 386)			
Hypersomnolence type, *n* (%)^†^						
Unable to confirm type	21 538 (94)	0	0			
IH	266 (1)	213 (19)	67 (17)	546	<.0001	B > C > A
Narcolepsy with cataplexy (NT1)	348 (2)	391 (34)	200 (52)	939	<.0001	C > B > A
Narcolepsy without cataplexy (NT2)	679 (3)	618 (54)	119 (31)	1416	<.0001	B > C > A
Sex, *n* (%)					<.0001	A > B,C
Male	14 422 (63)	646 (57)	208 (54)	15 276		
Female	8373 (37)	490 (43)	178 (46)	9041		
Birth month, *n* (%)					.6393	
March to May	5361 (24)	259 (23)	96 (25)	5716		
June to August	5493 (24)	276 (24)	101 (26)	5870		
September to November	6056 (27)	301 (26)	106 (27)	6463		
December to February	5885 (26)	300 (26)	83 (22)	6268		
Age in 2016,^‡^ years, mean (SD)	47.2 (19.2)	29.7 (15.7)	23.7 (10.3)		<.0001	A > B > C

*p*-values are for the comparison of the three groups. Group A: 3-digit ICD-9 code, with 347 for narcolepsy and 327 or 780.54 for hypersomnia; 3-digit ICD-10 code, with G47.4 for narcolepsy and G47.1 for hypersomnia. Group B: narcolepsy was identified using 5-digit ICD-10 codes (G47.411 or G47.421 for NT1, and G47.419 or G47.429 for NT2). IH was identified using ICD-10 codes (G47.11 and G47.12). Group C had strict diagnostic criteria: narcolepsy per the ICSD-3 criteria and following more than 1 year with two PSG and MSLT tests.

^*^Sleep study: either PSG or MSLT (National Health Insurance code: 20044B).

^†^Patients may have more than one type of hypersomnolence in the NHIRD.

^‡^Age at December 31, 2016.

ICD-9, International Classification of Diseases, Ninth Revision; ICD-10, International Classification of Diseases, 10th Revision; IH, idiopathic hypersomnia; MSLT, multiple sleep latency test; NHIRD, National Health Insurance Research Database; NT1, type 1 narcolepsy; NT2, type 2 narcolepsy; PSG, polysomnography; SD, standard deviation.

Use of stricter criteria for narcolepsy, defined as the presence of one or more ICD-10 codes and a sleep study within 6 months before or after the diagnosis date, resulted in lower narcolepsy prevalence estimates in the NHIRD cohort, increasing from 2.59 to 4.47 per 100 000 people between 2009 and 2019 (Table 2). Before 2016, most individuals diagnosed with central hypersomnia could not be further classified as having NT1, NT2, or IH owing to the ICD-9 coding system in use by the NHIRD. The NHIRD shifted from ICD-9 to ICD-10 in 2016, allowing classification into NT1, NT2, and IH. Consequently, annual prevalences and incidences for NT1, NT2, and IH could be calculated only for the period between 2016 and 2019, during which time the prevalence increased from 1.85 to 2.09 per 100 000 people for NT1 and from 1.70 to 2.38 per 100 000 for NT2, and decreased from 1.11 to 0.48 per 100 000 for IH (Table 2). Narcolepsy prevalence was higher in males versus females and highest in young adults aged 21–30 years (Supplementary Figure S1).

**Table 2.** Narcolepsy and IH Prevalence and Incidence in Taiwan (2009–2019) Based on the NHIRD

Year	Population at end of year, *n*	Narcolepsy		NT1		NT2		IH	
		*n*	Per 100 000 people	n	Per 100 000 people	*n*	Per 100 000 people	*n*	Per 100 000 people
Incidence	Total people								
2010	23 162 123	331	1.43						
2011	23 224 912	364	1.57						
2012	23 315 822	370	1.59						
2013	23 373 517	281	1.20						
2014	23 440 278	312	1.33						
2015	23 492 074	338	1.44						
2016	23 539 816	333	1.41						
2017	23 571 227	435	1.85	161	0.68	274	1.16	95	0.40
2018	23 588 932	357	1.51	117	0.50	240	1.02	72	0.31
2019	23 603 121	409	1.73	139	0.59	270	1.14	54	0.23
Prevalence	Total people								
2010	23 162 123	599	2.59						
2011	23 224 912	681	2.93						
2012	23 315 822	742	3.18						
2013	23 373 517	680	2.91						
2014	23 440 278	745	3.18						
2015	23 492 074	820	3.49						
2016	23 539 816	835	3.55	435	1.85	400	1.70	262	1.11
2017	23 571 227	1000	4.24	476	2.02	524	2.22	180	0.76
2018	23 588 932	975	4.13	445	1.89	530	2.25	148	0.63
2019	23 603 121	1055	4.47	493	2.09	562	2.38	113	0.48

Based on patients with central hypersomnia in the NHIRD with International Classification of Diseases codes for narcolepsy/IH.

IH, idiopathic hypersomnia; NHIRD, National Health Insurance Research Database; NT1, type 1 narcolepsy; NT2, type 2 narcolepsy.

The annual incidence of narcolepsy ranged from 1.20 to 1.85 per 100 000 people between the years 2010 and 2019, showing no overall trends over time (Table 2). The annual incidence of NT1 and NT2, defined by ICD-10 codes, remained stable between 2017 and 2019, while the incidence of IH decreased slightly from 0.40 per 100 000 in 2017 to 0.23 per 100 000 in 2019 (Table 2). Narcolepsy incidence was higher in males versus females in most years, except for 2017 and 2018, and was highest in children and young adults aged 0–30 years (Supplementary Figure S1).

### Validation of NT1 and NT2 diagnoses in the NHIRD

Patients with a confirmed diagnosis of narcolepsy from the gold-standard hospital database cohort were used to validate outcomes from the NHIRD database. Among 331 patients with narcolepsy from the hospital database, only 161 patients with NT1 and 29 patients with NT2 could be accurately identified in the NHIRD; thus, the precision of a narcolepsy diagnosis by the NHIRD was 57.4% (190/331).

### Burden of patients with NT1, NT2, and IH

We analyzed disease impact, including comorbidities, prescriptions, and healthcare utilization in both the NHIRD and hospital database cohorts separately. A total of 2606 patients with confirmed diagnoses (5-digit ICD-10 codes for NT1, NT2, or IH) were identified in the NHIRD and were included in analyses of disease impact (Figure 2). In addition, the disease burden of 386 patients with NT1, NT2, or IH from the hospital database cohort was tracked in the NHIRD as a comparison (Figure 2). Patients with NT1, NT2, or IH in the NHIRD and hospital database cohorts were compared with 5212 and 772 age- and sex- matched controls, respectively, extracted from the NHIRD (Figure 2).

In the NHIRD database, the proportion of patients with comorbidities was higher for those with NT1, NT2, or IH compared with the control group (all *p* ≤ .0001; Table 3). Comparisons between the NT1, NT2, and IH groups showed that a significantly higher proportion of patients with NT1 or NT2 had psychiatric comorbidities, including anxiety disorders, major depression, and schizophrenia, compared with those with IH (NT1, NT2 > IH; Table 3). Significantly more patients with NT1 had depression than those with NT2. Significantly more patients with NT2 had anxiety disorders, bipolar disorders, or ADHD than those with NT1 or IH, whereas the proportion of patients with ASD was higher with IH than with NT1. Other sleep disorders were significantly more frequent with NT1 or NT2 compared with IH, whereas obesity was significantly more frequent with IH and NT1 versus with NT2, and OSA, hypotension, and cardiovascular disease were significantly more frequent with IH than with NT1 or NT2.

**Table 3.** Demographic Characteristics and Comorbid Conditions in Patients With Narcolepsy or IH and the Control Group in the NHIRD Analysis (2016–2019)

	NHIRD	2016–2019 NHIRD				*P*1-value for 2 groups	*P*2-value for 4 groups^*^	Post hoc (groups)
	Sex- and age-matched group (*n* = 5212)	Total (IH + NT1 + NT2) (*n* = 2606)	IH (*n* = 459)	NT1 (*n* = 894)	NT2 (*n* = 1253)			
Sex, *n* (%)						.999	<.0001	0,2,3 < 1
Male	2860 (54.9)	1430 (54.9)	309 (67.3)	464 (51.9)	657 (52.4)			
Female	2352 (45.1)	1176 (45.1)	150 (32.7)	430 (48.1)	596 (47.6)			
Birth month, *n* (%)								
March to May	1194 (22.9)	623 (23.9)	133 (29.0)	212 (23.7)	278 (22.2)	.005	.095	
June to August	1295 (24.8)	636 (24.4)	109 (23.7)	226 (25.3)	301 (24.0)			
September to November	1445 (27.7)	688 (26.4)	110 (24.0)	246 (27.5)	332 (26.5)			
December to February	1278 (24.5)	659 (25.3)	107 (23.3)	210 (23.5)	342 (27.3)			
Age in 2016,^†^ years, mean (SD)	32.8 (18.0)	32.8 (18.0)	46.2 (20.2)	29.7 (15.6)	30.2 (16.4)	.9197	<.001	2,3 < 0 < 1
Comorbidity, *n* (%)								
Anxiety disorders	217 (4.2)	615 (23.6)	77 (16.8)	228 (25.5)	310 (24.7)	<.0001	<.0001	0,1 < 2,3
Major depression	126 (2.4)	581 (22.3)	65 (14.2)	198 (22.1)	318 (25.4)	<.0001	<.0001	0,1 < 2,3
Bipolar disorder	25 (0.5)	112 (4.3)	9 (2.0)	27 (3.0)	76 (6.1)	<.0001	<.0001	0 < 2,3; 1,2 < 3
Schizophrenia	33 (0.6)	79 (3.0)	5 (1.1)	38 (4.3)	36 (2.9)	<.0001	<.0001	0,1 < 2,3
ASD	6 (0.1)	22 (0.8)	6 (1.3)	5 (0.6)	11 (0.9)	<.0001	<.0001	0,2 < 1
ADHD	12 (0.2)	193 (7.4)	16 (3.5)	60 (6.7)	117 (9.3)	<.0001	<.0001	0 < 1,2 < 3
Epilepsy	8 (0.2)	101 (3.9)	18 (3.9)	39 (4.4)	44 (3.5)	<.0001	<.0001	0 < 1,2,3
Obstructive sleep apnea	15 (0.3)	333 (12.8)	97 (21.1)	93 (10.4)	143 (11.4)	<.0001	<.0001	0 < 2,3 < 1
Other sleep disorders	272 (5.2)	1108 (42.5)	191 (41.6)	391 (43.7)	526 (42.0)	<.0001	<.0001	0 < 1 < 2,3
Obesity	24 (0.5)	60 (2.3)	12 (2.6)	23 (2.6)	25 (2.0)	<.0001	<.0001	0 < 2
Diabetes mellitus	249 (4.8)	229 (8.8)	86 (18.7)	65 (7.3)	78 (6.2)	<.0001	<.0001	0 < 1,2
Hypertension	465 (8.9)	386 (14.8)	167 (36.4)	98 (11.0)	121 (9.7)	<.0001	<.0001	0,2,3 < 1
Hyperlipidemia	378 (7.3)	356 (13.7)	130 (28.3)	89 (10.0)	137 (10.9)	<.0001	<.0001	0 < 1
Cardiovascular disease	246 (4.7)	334 (12.8)	118 (25.7)	92 (10.3)	124 (9.9)	<.0001	<.0001	0,3 < 1,2
Cerebrovascular disease	55 (1.1)	89 (3.4)	35 (7.6)	23 (2.6)	31 (2.5)	<.0001	<.0001	0 < 1,2
Autoimmune diseases	119 (2.3)	99 (3.8)	24 (5.2)	33 (3.7)	42 (3.4)	.0001	.0016	0 < 1,2,3

*p*1-values are for the comparison of two groups (the NHIRD group and age- and sex-matched NHIRD controls). *p*2-values are for the comparison of four groups. Post hoc: 0 = NHIRD age- and sex-matched group, 1 = IH, 2 = NT1, 3 = NT2.

^*^Adjusted binary logistic regression model for age and sex categories as predictors for comorbidities and comedications.

^†^Age at December 31, 2016.

ADHD, attention-deficit/hyperactivity disorder; ASD, autism spectrum disorder/Asperger; IH, idiopathic hypersomnia; NHIRD, National Health Insurance Research Database; NT1, type 1 narcolepsy; NT2, type 2 narcolepsy; SD, standard deviation.

Although a higher proportion of patients with NT1, NT2, or IH from the hospital cohort had comorbidities than their matched controls, this analysis was limited due to a small sample size and the restriction of the NHIRD to protect privacy (case numbers of fewer than four are not shown); as a result, few significant differences between groups were observed (Table 4). Compared with the control group, more patients with NT1, NT2, or IH from the hospital database cohort had psychiatric comorbidities (significant differences were found for anxiety disorder and major depression, both *p* < .0001) and other comorbidities (significant differences were found for diabetes mellitus and cardiovascular disease [both *p* = .002], hyperlipidemia [*p* = .014], and other sleep disorders [*p* < .0001]). Although comparison with controls from the NHIRD was not applicable due to the forementioned restrictions, 3.9% of patients in the hospital cohort had schizophrenia (especially NT1, 6.5%), 9.8% had ADHD, 1.3% had ASD (especially NT2, 14.3%), and 8.8% had OSA (especially NT1 and NT2, 10.5% and 10.1%, respectively).

**Table 4.** Demographic Characteristics and Comorbid Conditions in Patients With Narcolepsy or IH and the Control Group in the Hospital Database Cohort

	NHIRD	Hospital cohort data (*n* = 386)				*P*1-value for 2 groups	*P*2-value for 4 groups^*^	Post hoc (groups)
	Sex- and age-matched group (*n* = 772)	Total (IH + NT1 + NT2) (*n* = 386)	IH (*n* = 67)	NT1 (*n* = 200)	NT2 (*n* = 119)			
Sex, *n* (%)						>.999	.346	—
Male	416 (53.9)	208 (53.9)	36 (53.7)	100 (50.0)	72 (60.5)			
Female	356 (46.1)	178 (46.1)	31 (46.3)	100 (50.0)	47 (39.5)			
Birth month, *n* (%)								
March to May	174 (22.5)	96 (24.9)	20 (29.9)	46 (23.0)	30 (25.2)	.761	.093	-
June to August	196 (25.4)	101 (26.2)	17 (25.4)	58 (29.0)	26 (21.8)			
September to November	223 (28.9)	106 (27.5)	18 (26.9)	63 (31.5)	25 (21.0)			
December to February	179 (23.2)	83 (21.5)	12 (17.9)	33 (16.5)	38 (31.9)			
Age in 2016, years,^†^ mean (SD)	23.7 (10.3)	23.7 (10.3)	27 (12.5)	23.2 (10.0)	22.4 (8.7)	.985	.005	0,2,3 < 1
Comorbidity, *n* (%)								
Anxiety disorders	24 (3.1)	114 (29.5)	6 (9.0)	76 (38.0)	32 (26.9)	<.0001	<.0001	0,1 < 2 < 3
Major depression	13 (1.7)	73 (18.9)	9 (13.4)	50 (25.0)	14 (11.8)	<.0001	<.0001	0,3 < 2
Bipolar disorder	—	6 (1.6)	1 (1.5)	3 (1.5)	2 (1.7)	—	—	—
Schizophrenia	—	15^‡^ (3.9)	1^‡^ (1.5)	13^‡^ (6.5)	1^‡^ (0.8)	—	—	—
ASD	—	5^‡^ (1.3)	1^‡^ (1.5)	2^‡^ (1.0)	2^‡^ (1.7)	—	—	—
ADHD	—	38^‡^ (9.8)	3^‡^ (4.5)	18^‡^ (9.0)	17^‡^ (14.3)	—	—	—
Obstructive sleep apnea	—	34^‡^ (8.8)	1^‡^ (1.5)	21^‡^ (10.5)	12^‡^ (10.1)	—	—	—
Other sleep disorders	24 (3.1)	178 (46.1)	13 (19.4)	102 (51.0)	63 (52.9)	<.0001	<.0001	0,1 < 2,3
Obesity	10 (1.3)	11 (2.8)	0^‡^ (0.0)	9^‡^ (4.5)	2^‡^ (1.7)	—	—	—
Diabetes mellitus	12 (1.6)	18 (4.7)	4 (6.0)	10 (5.0)	4 (3.4)	.002	.018	0 < 1,2
Hypertension	14 (1.8)	15^‡^ (3.9)	2^‡^ (3.0)	10^‡^ (5.0)	3^‡^ (2.5)	—	—	—
Hyperlipidemia	23 (3.0)	23 (6.0)	7 (10.4)	11 (5.5)	5 (4.2)	.014	.081	—
Cardiovascular disease	13 (1.7)	19 (4.9)	5 (7.5)	7 (3.5)	7 (5.9)	.002	.009	0 < 1,2; 3 < 1
Autoimmune diseases	12 (1.6)	14^‡^ (3.6)	2^‡^ (3.0)	6^‡^ (3.0)	6^‡^ (5.0)	—	—	—

*p*1-values are for the comparison of two groups (the hospital cohort group and controls). *p*2-values are for the comparison of four groups. Post hoc: 0 = NHIRD age- and sex-matched group, 1 = IH, 2 = NT1, 3 = NT2.

^*^Adjusted binary logistic regression model for age and sex categories as predictors for comorbidities and comedications.

^†^Age at December 31, 2016.

^‡^Case numbers fewer than four extracted from the NHIRD were prohibited to analyze. Data presented were from the hospital cohort without matching the NHIRD data.

ADHD, attention-deficit/hyperactivity disorder; ASD, autism spectrum disorder/Asperger; IH, idiopathic hypersomnia; NHIRD, National Health Insurance Research Database; NT1, type 1 narcolepsy; NT2, type 2 narcolepsy; SD, standard deviation.

Comparisons between NT1, NT2, and IH were similar for the NHIRD cohort (Table 4), with significant differences in the frequencies of anxiety disorders (higher for NT1 and NT2 vs IH), major depression (higher for NT1 vs NT2 and IH), other sleep disorders (higher for NT1 and NT2 vs IH), diabetes mellitus (higher for NT1 and IH vs NT2), hyperlipidemia (higher for IH than NT1 and NT2), and cardiovascular disease (higher for IH than NT1).

### Prescriptions for patients with NT1, NT2, and IH

#### Medications for hypersomnolence

In the NHIRD analysis (2606 patients with 5-digit ICD-10 codes for NT1, NT2, or IH), MPH and modafinil were prescribed to 44.7%, 0.4%, and 18.8% of patients with NT1, NT2, and IH, respectively, and 0% of controls (Table 5). Between groups, significantly more patients with NT1 received a prescription for modafinil than those with NT2 or IH, and significantly more patients with NT1 or NT2 received a prescription for MPH than those with IH. In the hospital database cohort, 43.3% and 54.9% of 386 patients with NT1, NT2, or IH received prescriptions for MPH and modafinil, respectively (Table 6); this is higher compared with the NHIRD. Between groups, more patients with NT1 and NT2 than IH received prescriptions for MPH (NT1, 53.5%; NT2, 44.5%; and IH, 10.4%) and modafinil (NT1, 77.5%; NT2, 46.2%; and IH, 3%).

**Table 5.** Medication Use, Healthcare Resource Utilization, and Healthcare Costs in the NHIRD Analysis (2016–2019)

	NHIRD	2016–2019 NHIRD				*P*1-value for 2 groups	*P*2-value for 4 groups^*^	Post hoc (groups)
	Sex- and age-matched group (*n *= 5212)	Total (IH + NT1 + NT2) (*n* = 2606)	IH (*n* = 459)	NT1 (*n* = 894)	NT2 (*n* = 1253)			
Treatment, *n* (%)								
Modafinil	0 (0.0)	492 (18.8)	3 (0.7)	291 (32.6)	198 (15.8)	<.0001	<.0001	1 < 3 < 2
Methylphenidate	19 (0.4)	1165 (44.7)	56 (12.2)	447 (50.0)	662 (52.8)	<.0001	<.0001	0,1 < 2,3
Comedications, *n* (%)								
Antidepressants	432 (8.3)	1192 (45.7)	159 (34.6)	449 (50.2)	584 (46.6)	<.0001	<.0001	0,1 < 2,3
Antipsychotics	781 (15.0)	932 (35.8)	131 (28.5)	299 (33.4)	502 (40.1)	<.0001	<.0001	0,1 < 2 < 3
Anxiolytics	1640 (31.5)	1476 (56.6)	285 (62.1)	480 (53.7)	711 (56.7)	<.0001	<.0001	0 < 1,2,3
Hypnotics and sedatives	440 (8.4)	742 (28.5)	150 (32.7)	252 (28.2)	340 (27.1)	<.0001	<.0001	0 < 2,3 < 1
Non-benzodiazepine-related drugs (zopiclone, zolpidem, zaleplon, eszopiclone)	235 (4.5)	457 (17.5)	93 (20.3)	148 (16.6)	216 (17.2)	<.0001	<.0001	0 < 1,2,3
Healthcare resource utilization, patients with ≥1 visit, *n* (%)								
Hospitalizations, patients with ≥ 1 admission	918 (17.6)	922 (35.4)	57 (56.0)	298 (33.3)	367 (29.3)	<.0001	<.0001	0,3 < 2 < 1
ED visits, patients with ≥1 visit	2258 (43.3)	1598 (61.3)	551 (120.0)	753 (84.2)	294 (23.5)	<.0001	<.0001	0 < 1,2
Healthcare costs, USD, median (IQR)								
All healthcare costs, inpatient and outpatient expenditure	113.18 (42.90–325.49)	710.86 (302.48–1729.81)	729.65 (314.94–1895.86)	879.33 (334.79–2718.56)	615.16 (271.37–1425.09)	<.0001	<.0001	0 < 1,3 < 2
Narcolepsy-related healthcare costs^†^	N/A	47.15 (5.20–163.66)	0 (0–0)	106.23 (35.16–586.98)	56.73 (16.92–140.29)	—	—	—

*p*1-values are for the comparison of two groups (the NHIRD group and controls). *p*2-values are for the comparison of four groups. Post hoc: 0 = NHIRD age- and sex-matched group, 1 = IH, 2 = NT1, 3 = NT2.

^*^Adjusted binary logistic regression model for age and sex categories as predictors for comorbidities, comedications, healthcare resource utilization, and costs.

^†^Costs of healthcare visits with narcolepsy codes, including sleep studies, medication, and clinical evaluation.

ED, emergency department; IH, idiopathic hypersomnia; IQR, interquartile range; N/A, not applicable; NHIRD, National Health Insurance Research Database; NT1, type 1 narcolepsy; NT2, type 2 narcolepsy; USD, United States dollars.

**Table 6.** Medication Use, Healthcare Resource Utilization, and Healthcare Costs in the Hospital Database Cohort

	NHIRD	Hospital cohort clinical data (*n* = 386)				*P*1-value for 2 groups	*P*2-value for 4 groups^*^	Post hoc (groups)
	Sex- and age-matched group (*n* = 772)	Total (IH + NT1 + NT2) (*n* = 386)	IH (*n* = 67)	NT1 (*n* = 200)	NT2 (*n* = 119)			
Treatment, *n* (%)								
Modafinil	—	212^†^ (54.9)	2^†^ (3.0)	155^†^ (77.5)	55^†^ (46.2)			—
Methylphenidate	—	167^†^ (43.3)	7^†^ (10.4)	107^†^ (53.5)	53^†^ (44.5)			—
Comedications, *n* (%)								
Antidepressants	26 (3.4)	159 (41.2)	13 (19.4)	117 (58.5)	29 (24.4)	<.0001	<.0001	0,1,3 < 2
Antipsychotics	24 (3.1)	100 (25.9)	13 (19.4)	54 (27.0)	33 (27.7)	<.0001	<.0001	0 < 2,3
Anxiolytics	59 (7.6)	150 (38.9)	28 (41.8)	79 (39.5)	43 (36.1)	<.0001	<.0001	0 < 1,2,3
Hypnotics and sedatives	26 (3.4)	54 (14.0)	9 (13.4)	34 (17.0)	11 (9.2)	<.0001	<.0001	0 < 2
Non-benzodiazepine-related drugs (zopiclone, zolpidem, zaleplon, eszopiclone)	18 (2.3)	37 (9.6)	8 (11.9)	21 (10.5)	8 (6.7)	<.0001	<.0001	0 < 2
Healthcare resource utilization, patients with ≥ 1 visit, *n* (%)								
Hospitalizations	103 (13.3)	85 (22.0)	16 (23.9)	44 (22.0)	25 (21.0)	.0003	.0026	0 < 1,2,3
ED visits	364 (47.2)	202 (52.3)	37 (55.2)	99 (49.5)	66 (55.5)	.105	.235	—
Healthcare costs, USD, median (IQR)								
All healthcare costs, inpatient and outpatient expenditure	96.60 (45.50–223.14)	1545.86 (330.23–3968.48)	274.43 (89.74–728.95)	3358.59 (1210.30–4716.24)	800.97 (209.44–2706.99)	<.0001	<.0001	0,1 < 2 < 3
Narcolepsy-related healthcare costs^‡^	N/A	149.65 (30.60–1890.66)	0 (0–0)	1470.44 (161.34–2299.99)	0 (0–378.12)	—	—	—

*p*1-values are for the comparison of two groups (the hospital cohort and controls). *p*2-values are for the comparison of four groups. Post hoc: 0 = NHIRD age- and sex-matched group, 1 = IH, 2 = NT1, 3 = NT2.

^*^Adjusted binary logistic regression model for age and sex categories as predictors for comorbidities, comedications, healthcare resource utilization, and costs.

^†^Case numbers fewer than four extracted from the NHIRD were prohibited to analyze. Data presented were from the hospital cohort without matching the NHIRD data.

^‡^Costs of visits with narcolepsy codes, including sleep studies, medication, and clinical evaluation.

ED, emergency department; NHIRD, National Health Insurance Research Database; IH, idiopathic hypersomnia; N/A, not applicable; NT1, type 1 narcolepsy; NT2, type 2 narcolepsy; USD, United States dollars.

#### Comedications

In the NHIRD database analysis, a significantly higher proportion of the 2606 evaluated patients with NT1, NT2, or IH received comedications than controls, including antidepressants (45.7% vs 8.3%), antipsychotics (35.8% vs 15.0%), anxiolytics (56.6% vs 31.5%), hypnotics and sedatives (28.5% vs 8.4%), and non-benzodiazepine–related Z-drugs (zopiclone, zolpidem, zaleplon, eszopiclone; 17.5% vs 4.5%; all *p* < .0001; Table 5). Comparison between diagnoses showed that a higher proportion of patients with NT1 or NT2 received antidepressant (NT1, NT2 > IH) and antipsychotic (NT1 > NT2 > IH) prescriptions than those with IH, and a higher proportion of patients with IH received prescriptions for hypnotics and sedatives than those with narcolepsy (IH > NT1, NT2). The analysis of comedications from the 386 evaluated patients from the hospital database revealed similar findings (all *p* < .0001; Table 6). A significantly higher proportion of patients with NT1 received a prescription for an antidepressant than those with NT2 or IH (*p* < .0001 for both comparisons).

### Healthcare resource utilization

Evaluated patients with NT1, NT2, or IH identified in the NHIRD analysis (*n = *2606) had higher healthcare resource utilization than controls (*n = *5212). This included the proportion of patients with at least one hospitalization (35.4% vs 17.6%) or emergency department visit (61.3% vs 43.3%) and all healthcare costs (710.86 USD vs 113.18 USD; all *p* < .0001; Table 5). Similar findings were found in the hospital cohort database analysis for NT1, NT2, and IH (*n = *386) versus controls (*n = *772), including significantly more hospitalizations (22.0% vs 13.3%; *p* = .0003) and higher healthcare costs (1545.86 USD vs 96.60 USD; *p* < .0001).

When comparing NT1, NT2, and IH diagnoses, the NHIRD analysis found that patients with IH had significantly more hospitalizations than those with NT1 or NT2 (*p* < .0001); however, patients with NT1 had higher medication use (modafinil or MPH) and higher healthcare costs than those with NT2 or IH (*p* < .0001; NT1 > NT2, IH). In the hospital database cohort, patients with NT2 had the highest costs compared with NT1 and IH (*p* < .0001; NT2 > NT1 > IH).

## Discussion

This study marks the first comprehensive investigation into the prevalence, incidence, and disease burden of narcolepsy and IH in Taiwan, leveraging data from two databases and corroborating data from the NHIRD with the hospital cohort database. Although the NHIRD offers a wealth of data, covering approximately 99% of Taiwan’s population, its analysis is not without limitations, including potential issues such as missing data, misdiagnosis, miscoding, and the absence of objective testing tools. Thus, comparison with the hospital database cohort served to enhance the credibility of our findings.

Global narcolepsy prevalence estimates are mostly between 20 and 50 per 100 000 people [16–24, 27, 28]. Although ethnic differences may contribute to the wide range, methodology differences can be a major reason why prevalence varies greatly around the world. The significant variations across studies mirror the diverse prevalence estimates obtained in our study with different inclusion criteria (100.82/100 000 for unspecified central hypersomnia diagnoses excluding patients with IH, and 9.98/100 000 for patients with NT1 or NT2 only). Notably, our more strict inclusion criteria yielded a prevalence of 9.98 per 100 000 (0.01%) in Taiwan from 2009 to 2019, which falls below estimates reported in other regions such as Hong Kong (0.034% [34/100 000]) [24], Europe (47/100 000), and the United States (38.9–79.4/100 000) [19, 20, 22, 28, 35, 57–60].

Besides methodological and ethnic differences, environmental factors, such as lifestyle differences and variations in healthcare and diagnostic systems, may influence prevalence estimates. In addition to the extensive coverage provided by Taiwan’s National Health Insurance, the country has 27 medical centers, 88 regional hospitals, and 350 district hospitals across a 35 886 km² area, making medical services accessible and inclusive. Nevertheless, only 58 (12.5%) of these hospitals have sleep centers that offer access for sleep studies [61]. The referral system in Taiwan operates less efficiently compared to other healthcare systems, as patients are not required to see a general practitioner for a referral. While this increases convenience, it may lead to patients consulting an inappropriate specialist who lacks knowledge of narcolepsy, resulting in inadequate evaluation and treatment. Physicians with limited clinical experience of narcolepsy and IH may be less alert to the possibility of a narcolepsy/IH diagnosis, and may arrange unnecessary examination for patients with daytime sleepiness without comprehensive clinical evaluation, wasting resources for accurate diagnosis. Furthermore, key diagnostic tests, such as PSG and MSLT and orexin levels, are not always available to clinicians; therefore, accurate diagnoses of narcolepsy or IH may not be possible without access to appropriate specialist physicians. We suggest that education of non-specialist healthcare practitioners about narcolepsy and IH is necessary for referral, diagnosis and early treatment. Additionally, setting compliant rules for the referral system and the hierarchy of medical services can guide patients and physicians to appropriate evaluations and efficiently utilize the resources of diagnostic tools, with the added effect of improving knowledge about the prevalence and incidence of narcolepsy.

Sleep efficiency secondary to overtime work and study is common in modern societies, which can influence the diagnosis and management of narcolepsy. Studies investigating subjective sleep and quality of life of patients with narcolepsy in Taiwan showed improvements during the COVID-19 lockdown periods [62, 63], which could be related to longer sleeping hours and a more flexible schedule for most people. Furthermore, people with undiagnosed narcolepsy may mistakenly attribute their symptoms to work or study burden, rather than to a sleep disorder. The general public may have limited awareness of the condition, and label those with narcolepsy or IH as lazy or depressed. Those with mild symptoms may adjust their lifestyle to cope with their symptoms instead of seeking a diagnosis or treatment. The observed discrepancy in prevalence underscores the importance of raising awareness among the public and improving diagnostic practices of clinicians for narcolepsy. The socio-cultural factors also highlight the governments’ regulation on work and school hours to decrease negative impacts on these patients.

Comparison of our results with the latest national database studies from Japan and South Korea showed that our estimates of annual narcolepsy prevalence and incidence in Taiwan are relatively lower than those in Japan and similar to those in South Korea. Compared with 4.47 and 1.72 per 100 000 in Taiwan (Supplementary Table S2), the annual prevalence and incidence of narcolepsy in South Korea were 8.4 and 1.3 per 100 000 in 2019 [64], respectively, while in two studies in Japan, prevalence estimates were 18.5 and 37.5 per 100 000 and incidence estimates were 4.3 and 5.1 per 100 000 [25, 26]. Similarly, our estimate for the prevalence of IH was lower than a recent claims-based study in Japan (2.3/100 000 in our study vs 7.7/100 000 in the Japanese study) [26]. As with findings from Western countries [20, 35], trends of increased incidence and prevalence over time were observed, especially in the young adult group. This increase may partially stem from changes in diagnostic coding systems (from the ICD-9 to ICD-10 codes), or to other factors, such as heightened recognition of narcolepsy and IH in the general population, which contributes to early detection in the young adult population. Furthermore, there may be increasing recognition related to the promotion of new medications for hypersomnolence.

We found that the prevalence and incidence were lower for NT1 than for NT2 in the NHIRD analysis over the evaluated period, with the exception of 2016, which coincides with the year in which the ICSD-3 was adopted in Taiwan. Although there is evidence supporting higher prevalence and incidence of NT2 than NT1 [20, 65], NT2 can be more difficult to diagnose than NT1 [57, 66], and clinical studies including our hospital cohort have reported opposite findings [32, 67]. Compared with NT1, which is a well-defined syndrome, patients with NT2 can have clinical and test variability over time [54]. These inconsistencies highlight the difficulties in the differential diagnosis of narcolepsy subtypes and the need to refine the diagnostic methods and criteria for NT2. Clinicians should be cautious in diagnosing NT2, considering that current diagnostic methods for the differential diagnosis are still not optimal. Routine follow-up sleep studies can provide information on treatment response and changes in disease severity, and thus should be considered every 1 to 2 years after initiating treatment for all patients with central hypersomnia disorders, especially in those with NT2 and IH.

Consistent with global trends [35, 39, 42, 68–70], our study revealed a significant burden of comorbidities, including psychiatric conditions, among patients with narcolepsy and IH, along with elevated healthcare utilization and costs. Common psychiatric comorbidities in patients with NT1, NT2, or IH included anxiety, major depression, bipolar disorder, schizophrenia, ASD, and ADHD. Additional common comorbidities included epilepsy, obesity, diabetes mellitus, hypertension, hyperlipidemia, cardiovascular disease, cerebrovascular disease, autoimmune diseases, OSA, and other sleep disorders. Of note, fewer comorbidities were found in the hospital database cohort. However, a substantial number of comorbidities were still identified in these patients. Psychiatric comorbidities, such as depression and anxiety, may be related to the distress resulting from narcolepsy, and a number of the additional common comorbidities may be caused by obesity associated with narcolepsy or orexin deficiency. It is also essential for health policy across different countries to lay emphasis on their assessment and treatment to decrease negative impacts on patients’ adjustment.

Trends of disease burden of central hypersomnia disorders were similar between the NHIRD and the hospital cohort database, but discrepancies were noted in a post hoc analysis of different central hypersomnia diagnoses. The NHIRD analysis showed no differences in depression and anxiety between patients with NT1 and NT2, whereas the hospital cohort database showed that significantly more patients with NT1 had depression than those with NT2, and patients with NT2 had more anxiety disorders than those with NT1 and IH. Previous studies are also inconsistent in the prevalence of depressive disorders between narcolepsy subtypes and IH. While some reported significantly more depression in patients with NT1 than with NT2 and IH [71, 72], others reported no difference between narcolepsy subtypes [73]. Comparison of comorbid anxiety between narcolepsy subtypes is similarly lacking. However, understanding the prevalence of these comorbidities and the effects of the conditions and their treatments on the symptoms of narcolepsy and IH can help inform patient management decisions. Both depression and anxiety can impact quality of life and may impair the ability of patients with narcolepsy to develop coping strategies to manage their condition. Their treatments often involve medications with sedative effects, including benzodiazepines and antidepressants such as paroxetine, which can negatively increase hypersomnolence. Research is required to investigate the associations between the two most common psychiatric disorders and narcolepsy subtypes and develop disease-specific treatment approaches.

Consistent with previous research [69, 74, 75], we found that patients with narcolepsy and IH had more obesity, metabolic disease, and cardiovascular disease than controls. Interestingly, more patients with IH had hypertension and cardiovascular disease than those with narcolepsy in the analysis of both the NHIRD and the hospital cohort database. Patients with IH often have autonomic nerve symptoms [76], and autonomic nerve dysfunction can have impacts on blood pressure and cardiovascular function [77]. Besides, the severity of excessive daytime sleepiness related to sleep disorders can correlate with cardiovascular risk [78], but no previous study compares the risk between different central hypersomnia disorders, highlighting the need for further investigation.

Annual medical resource utilization and medical expenses of patients with NT1, NT2, or IH were significantly higher than in controls, especially in those with NT1 or NT2. These findings provide insight into comorbidities associated with narcolepsy, which can be found even in young patients. Patients with NTI had the highest healthcare costs in the hospital cohort database, close to those of patients with malignancy, such as breast cancer [79], whereas patients with NT2 had the highest costs in the NHIRD. Although patients with NT1 are assumed to have higher healthcare costs considering symptom severity and comorbidities, high costs of patients with NT2 and high rates of admission and ER visits of patients with IH in the NHIRD can be contributed to by other medical conditions. For instance, research indicates that patients with NT2 exhibit the highest risk of accidents and injuries, followed by patients with IH, while those with NT1 demonstrate the lowest risk [80]. Interventions including risk prevention and pharmacoeducation to enhance adherence should be provided to all patients with central hypersomnia disorders. Besides, the healthcare costs of NT1 from the NHIRD analysis were much lower than those from the hospital cohort database, and the increased rates of hospitalization and ER visits of patients with IH and NT2 were not observed in the hospital cohort. Considering the significant differences in age and sex, these discrepancies in comorbidity rate and healthcare costs between the NHIRD and hospital cohort data also underscore the impact of sample demographics and limitations of the two databases, and emphasize the need for tailored management strategies for patients with different central hypersomnia diagnoses.

It is interesting that fewer patients from the NHIRD were treated with prescription medications for hypersomnolence compared with those from the hospital cohort, but many were treated with concomitant comorbidities, including antidepressants, antipsychotics, anxiolytics, and hypnotics. The findings indicate that a significant proportion of patients with central hypersomnia may not receive appropriate treatment after diagnosis. In Taiwan, patients treated with modafinil must receive annual PSG and MSLT, which can represent a treatment barrier. Those who do not receive these annual exams are not permitted to receive the modafinil prescription and can only take methylphenidate for hypersomnolence. This may explain why fewer patients from the NHIRD were treated with modafinil than in the hospital database cohort, and that more patients with NT2 received methylphenidate in the NHIRD compared to those in the hospital cohort database. Sleep studies are time and labor-consuming, and waiting lists are long; therefore, patients may be unable to receive these exams, accounting for the lower prescribing prevalence. Identifying barriers to accessing medical treatments and implementing case management strategies for severe patients may prove helpful to inform improvements in care.

Considering the high rates of comorbid depression and anxiety, and the need to treat cataplexy, it is not surprising that antidepressant and anxiolytic prescriptions were common in patients from both the NHIRD and the hospital cohort databases. In addition, more than 25% of patients from our hospital database cohort received antipsychotics, which were prescribed to treat hallucinations related to narcolepsy, psychotic symptoms related to comorbid schizophrenia, and as an add-on therapy for depression. An even higher percentage of patients were prescribed an antipsychotic prescription (up to 35.8% in the NHIRD cohort), which is in line with epidemiological findings of trends of increasing antipsychotic use [81]. When prescribing psychotropics, clinicians should consider side effects and metabolic risk [82], as metabolic diseases and obesity are common in patients with central hypersomnia.

We found that hypnotic and Z-drugs were commonly prescribed for patients with narcolepsy and IH. Although excessive daytime sleepiness is always present in patients with narcolepsy, up to 60.8% of untreated and 40.3% of treated patients with narcolepsy have disturbed nighttime sleep [83]. Patients with narcolepsy can have increased awakenings and arousals, increased percentage of stage 1 sleep, decreased percentage of stage 2 sleep, and decreased sleep efficiency as measured by PSG compared with healthy populations [84]. Decreased total sleep time, more wake time after sleep onset, more awakenings, and more sleep motor activity were also found in patients with narcolepsy, using actigraphy, versus healthy populations [85, 86]. Additionally, the medication of choice for disturbed nighttime sleep is sodium oxybate [87–89], which is not available in Taiwan. All of these conditions can be the causes for increased hypnotic and Z-drug use. Although a guideline has suggested benzodiazepines and non-benzodiazepine hypnotics for disturbed nighttime sleep [90], none are approved by the US Food and Drug Administration or the European Medicines Agency. Clinicians must exercise caution in prescribing sedative medications, considering the risk of exacerbating hypersomnolence and related accident or injury.

### Limitations

Despite comparison with a hospital database cohort, several limitations to these analyses warrant consideration. First, the inherent limitations of claim databases, including missing data, privacy restrictions, and diagnostic inaccuracies, pose challenges to data interpretation. The precision of narcolepsy diagnosis was found to be 57.4%, which means nearly half of the patients with narcolepsy identified in the hospital database cohort could not be accurately identified in the NHIRD. These findings suggest that narcolepsy prevalence estimates from this study may be underestimates. Other diagnoses extracted from the NHIRD have also been validated, and the results varied from modest to high sensitivity [52]. The prevalence of narcolepsy itself can play a role. Diseases that are common can be coded more often in the NHIRD and thus have better precision. Considering the low prevalence of central hypersomnia, the number of patients may be underestimated. In addition, discrepancies in diagnostic coding systems and the exclusion of self-paid healthcare from the NHIRD further impact data accuracy.

## Conclusion

Our study reveals a lower prevalence of narcolepsy and IH in Taiwan compared with other countries, indicating potential underdiagnosis. The discrepancy in medication prescriptions between the NHIRD and the hospital database cohort underscores the need for improved treatment. The high proportion of patients with comorbidities and substantial healthcare utilization reminds us of the substantial disease impact of central hypersomnia.

## Supplementary Material

zsaf132_suppl_Supplementary_Tables_S1-S2_Figure_S1

## Data Availability

Anonymized data that support the findings of this study are available on reasonable request from the corresponding author, Wei-Chih Chin.
