# Supplementary material for: The prevalence, incidence, and impact of narcolepsy and idiopathic hypersomnia in Taiwan: comparison between the National Health Insurance Research Claims Database and a hospital cohort database
Source: Sleep. 2025 May 20;48(11):zsaf132. doi: 10.1093/sleep/zsaf132 (PMC12597673; doi:10.1093/sleep/zsaf132)
Supplement: zsaf132_suppl_Supplementary_Tables_S1-S2_Figure_S1 [file zsaf132_suppl_supplementary_tables_s1-s2_figure_s1.docx]

**Supplementary Material**

**The prevalence and burden of central disorders of hypersomnolence in Taiwan: comparison between the National Health Insurance Research Claims Database and a hospital cohort database**

Yu-Shu Huang^1,2^, Wei-Chih Chin^1,2,3*^, I-Hang Chung^1,2^, Tsun-Yi Roan^1,2^, Chee-Jen Chang^4,5,6^, Hsiao-Ting Juang^4^, Shu-Chen Chang^5^, Somraj Ghosh^7^, Stephen Crawford^7^ and Huang-Li Lin^1,2^

^1^Division of Psychiatry and Sleep Center, Chang Gung Memorial Hospital, Taoyuan, Taiwan

^2^College of Medicine, Chang Gung University, Taoyuan, Taiwan

^3^College of Life Sciences and Medicine, National Tsing Hua University, Hsinchu, Taiwan

^4^Department of Artificial Intelligence, Chang Gung University, Taoyuan, Taiwan

^5^Research Services Center for Health Information, Chang Gung University, Taoyuan, Taiwan

^6^Department of Biomedical Sciences, Chang Gung University, Taoyuan, Taiwan

^7^Takeda Development Center Americas, Inc., Cambridge, MA, USA

*Corresponding author. Wei-Chih Chin, Department of Psychiatry, Chang Gung Memorial Hospital, No. 5, Fuxing St., Guishan Dist., Taoyuan City 333, Taiwan. Phone: 886-3-3281200 ext. 2479. Fax: 886-3-3280267. E-mail: [auaug0327@hotmail.com](mailto:auaug0327@hotmail.com)

**Table of contents**

**Supplementary Figure S1.** Annual narcolepsy prevalence and incidence rates in Taiwan from 2009 to 2019 identified from the National Health Insurance Research Database analysis, overall and according to sex and age group. 2

**Supplementary Table S1.** Case Definitions for Narcolepsy and Idiopathic Hypersomnia 3

**Supplementary Table S2.** Comparison of Prevalence and Incidence Rates of Narcolepsy Among Taiwan, Japan, and South Korea 4

**References** 4

# Supplementary Figure S1. Annual narcolepsy prevalence and incidence rates in Taiwan from 2009 to 2019 identified from the National Health Insurance Research Database analysis, overall and according to sex and age group.


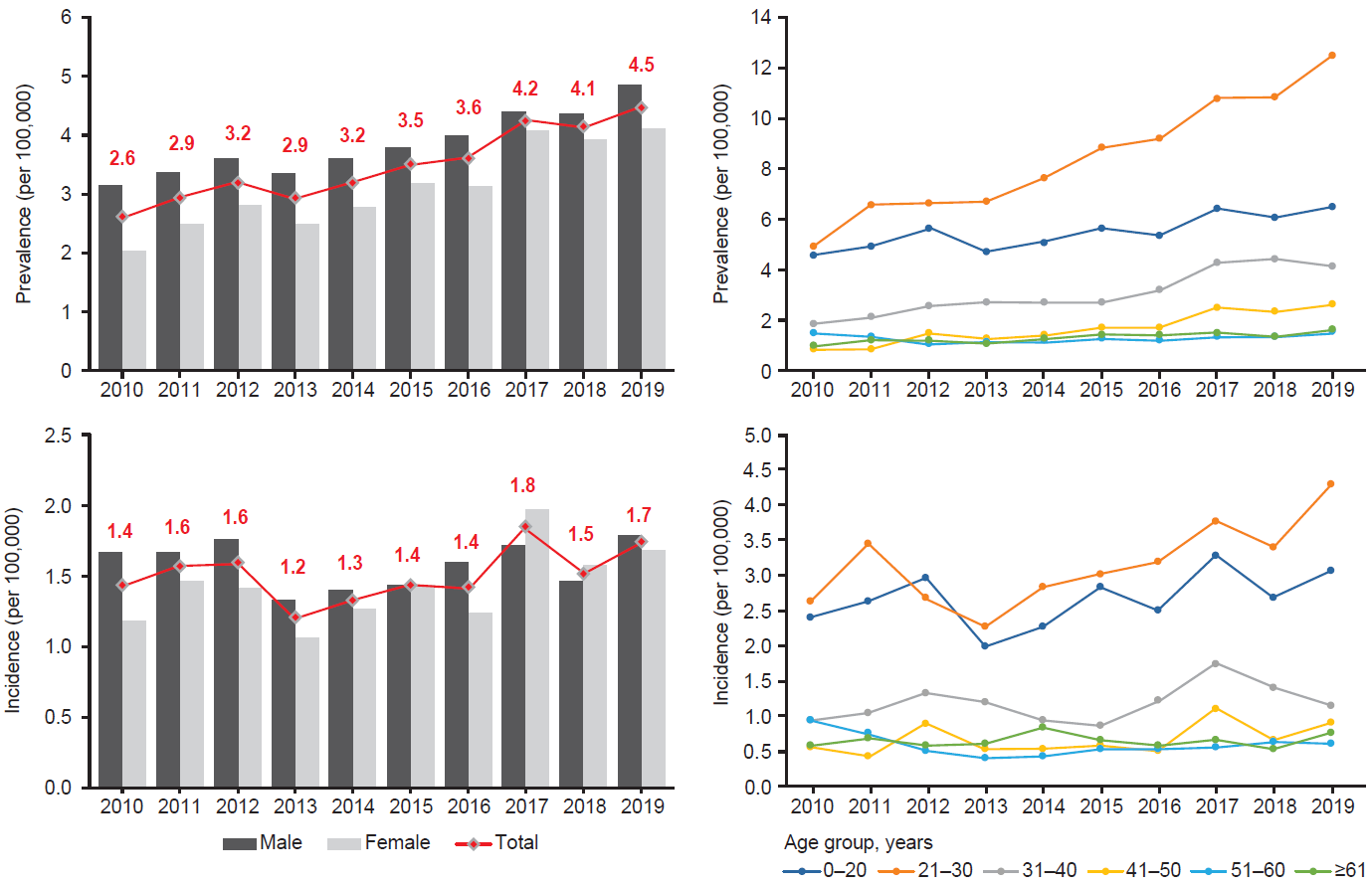


# Supplementary Table S1. Case Definitions for Narcolepsy and Idiopathic Hypersomnia

| **Type** | **ICD version** | **ICD code** | **Definition** |
| --- | --- | --- | --- |
| NT1 | ICD-9-CM | 347.01 | Narcolepsy, with cataplexy |
| NT1 | ICD-9-CM | 347.11 | Narcolepsy in conditions classified elsewhere, with cataplexy |
| NT1 | ICD-10-CM | G47.411 | …… with cataplexy |
| NT1 | ICD-10-CM | G47.421 | …… with cataplexy |
| NT2 | ICD-9-CM | 347.00 | Narcolepsy, without cataplexy |
| NT2 | ICD-9-CM | 347.10 | Narcolepsy in conditions classified elsewhere, without cataplexy |
| NT2 | ICD-10-CM | G47.419 | …… without cataplexy |
| NT2 | ICD-10-CM | G47.429 | …… without cataplexy |
| IH | ICD-9-CM | 327.11 | Idiopathic hypersomnia with long sleep time |
| IH | ICD-9-CM | 327.12 | Idiopathic hypersomnia without long sleep time |
| IH | ICD-10-CM | G47.11 | Idiopathic hypersomnia with long sleep time |
| IH | ICD-10-CM | G47.12 | Idiopathic hypersomnia without long sleep time |

ICD-9-CM, International Classification of Diseases, Ninth Revision, Clinical Modification; ICD-10-CM, International Classification of Diseases, 10th Revision, Clinical Modification; IH, idiopathic hypersomnia; NT1, type 1 narcolepsy; NT2, type 2 narcolepsy.

# Supplementary Table S2. Comparison of Prevalence and Incidence Rates of Narcolepsy Among Taiwan, Japan, and South Korea

| **Prevalence** | **Japan*** | **Korea^†^** | **Taiwan^‡^** | **Incidence** | **Japan*** | **Korea^†^** | **Taiwan^‡^** |
| --- | --- | --- | --- | --- | --- | --- | --- |
| **2010** | 5.7 |  | 2.59 | **2010** | 3.6 | 0.77 | 1.43 |
| **2011** | 7.3 |  | 2.93 | **2011** | 3.1 | 0.85 | 1.57 |
| **2012** | 6.8 |  | 3.18 | **2012** | 3.2 | 0.90 | 1.59 |
| **2013** | 11.3 |  | 2.91 | **2013** | 4.4 | 0.81 | 1.20 |
| **2014** | 11.2 | 4.7 | 3.18 | **2014** | 3.6 | 1.06 | 1.33 |
| **2015** | 15.0 | 5.5 | 3.49 | **2015** | 4.5 | 1.03 | 1.44 |
| **2016** | 15.1 | 6.2 | 3.55 | **2016** | 4.9 | 1.17 | 1.44 |
| **2017** | 16.3 | 7.0 | 4.24 | **2017** | 4.5 | 1.38 | 1.85 |
| **2018** | 17.4 | 7.9 | 4.13 | **2018** | 3.3 | 1.36 | 1.50 |
| **2019** | 18.5 | 8.4 | 4.47 | **2019** | 4.3 | 1.28 | 1.72 |

*Records of narcolepsy diagnosis as ICD-10 code (G474) in ≥2 consecutive months during the study period in an employment-based health insurance claims database compiled by JMDC Inc. [1].

^†^Annual trend of definite narcolepsy (estimated from the Rare and Intractable Disease Register) [2].

^‡^The primary and secondary diagnoses code by ICD-9 (347) and ICD-10 (G474) from outpatient departments from the Taiwanese National Health Insurance Research Database.

ICD-9, International Classification of Diseases, Ninth Revision; ICD-10, International Classification of Diseases, 10th Revision.

# References

1. Imanishi A, Kamada Y, Shibata K, Sakata Y, Munakata H, Ishii M. Prevalence, incidence, and medications of narcolepsy in Japan: a descriptive observational study using a health insurance claims database. *Sleep Biol Rhythms.* 2022;**20**(4):585-594. doi: 10.1007/s41105-022-00406-4.

2. Park HR, Song P, Lee SY, Epidemiology Committee of Korean Sleep Research. National estimates of narcolepsy in Korea. *J Clin Neurol.* 2023;**19**(1):83-89. doi: 10.3988/jcn.2023.19.1.83.
